# Cells in Atherosclerosis: Focus on Cellular Senescence from Basic Science to Clinical Practice

**DOI:** 10.3390/ijms242417129

**Published:** 2023-12-05

**Authors:** Andrea Ágnes Molnár, Dorottya Tímea Pásztor, Zsófia Tarcza, Béla Merkely

**Affiliations:** Heart and Vascular Center, Semmelweis University, 1122 Budapest, Hungary; pasztor.dorottya.timea@gmail.com (D.T.P.); tarczazsofi@gmail.com (Z.T.); merkely.bela@gmail.com (B.M.)

**Keywords:** cellular senescence, atherosclerosis, oxidative stress, inflammation, senotherapeutics

## Abstract

Aging is a major risk factor of atherosclerosis through different complex pathways including replicative cellular senescence and age-related clonal hematopoiesis. In addition to aging, extracellular stress factors, such as mechanical and oxidative stress, can induce cellular senescence, defined as premature cellular senescence. Senescent cells can accumulate within atherosclerotic plaques over time and contribute to plaque instability. This review summarizes the role of cellular senescence in the complex pathophysiology of atherosclerosis and highlights the most important senotherapeutics tested in cardiovascular studies targeting senescence. Continued bench-to-bedside research in cellular senescence might allow the future implementation of new effective anti-atherosclerotic preventive and treatment strategies in clinical practice.

## 1. Introduction

**Figure 1:**
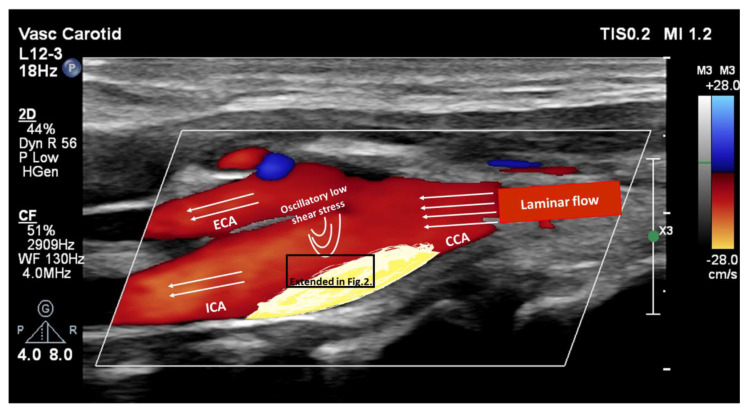
Carotid Duplex ultrasound color image of carotid artery bifurcation superimposed with a schematic illustration of blood flow and carotid atherosclerotic plaque. The laminar blood flow becomes oscillatory at the sites of bifurcation, which promotes the initiation of atherosclerotic plaque formation. The black rectangle on the atherosclerotic plaque represents the extended site seen in Figure 2. CCA: common carotid artery, ICA: internal carotid artery, ECA: external carotid artery.

**Figure 2:**
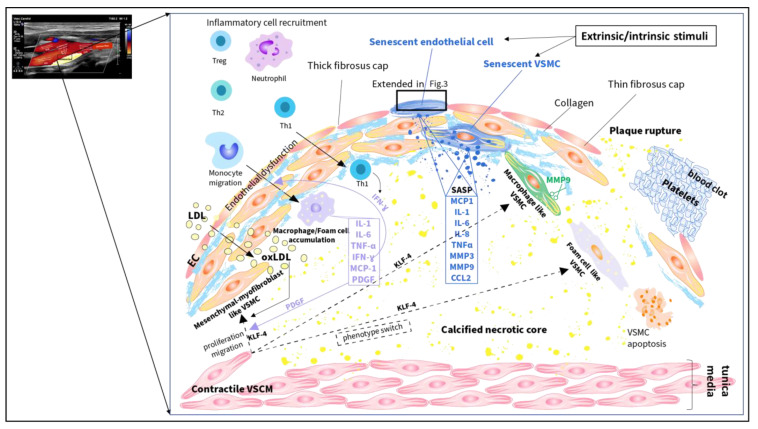
Schematic illustration of cellular alterations in atherosclerosis. Extracellular and intracellular stimuli can promote cellular senescence and endothelial dysfunction facilitating low-density lipoprotein (LDL) and immune cell infiltration into the subendothelial space. In the subendothelial space, the monocytes differentiate into macrophages consuming lipids and form foam cells, which leads to lipid core development. The level of inflammatory cytokines, chemokines, growth factors, and matrix metalloproteinases (MMP) increases. Platelet-derived growth factor (PDGF) induces phenotype switch of the contractile vascular smooth muscle cells (VSMCs) to mesenchymal-like VSMCs, which can further shift to myofibroblast-like VSMCs. Inflammatory cytokines and oxidized LDL (ox-LDL) also stimulate the contractile VSMC to myofibroblast-like VSMC transition and the migration and proliferation of these cells from tunica media to tunica intima. These cells produce extracellular matrices (such as collagen), which leads to the formation of fibrous caps in the advanced phase. In addition, the contractile VSMCs can switch to macrophage-like and foam cell-like VSMCs. All VSMC phenotype switches can be induced by Krüppel-like factor 4 (KLF4). Extracellular matrix degradation and reduced collagen synthesis lead to fibrous cap thinning mainly due to both endothelial cell (EC) and VSMC-derived senescence-associated secretory phenotype (SASP) senescent cells, macrophage-like VSMCs and neutrophil cells releasing MMPs. Plaques with thin fibrous caps are prone to rupture, which triggers blood clot formation. The black rectangle on the senescent cell represents the extended site seen in Figure 3. IL-1: interleukin-1, IL-6: interleukin-6, IL-8: interleukin-8, TNF-α: tumor necrosis factor-α, IFN-γ: interferon-γ, MCP-1: monocyte chemokine protein-1, CCL-2: C-C motif chemokine ligand 2, Th1: T-helper1 lymphocyte, Th2: T-helper2 lymphocyte, Treg: regulatory T lymphocyte.

**Figure 3:**
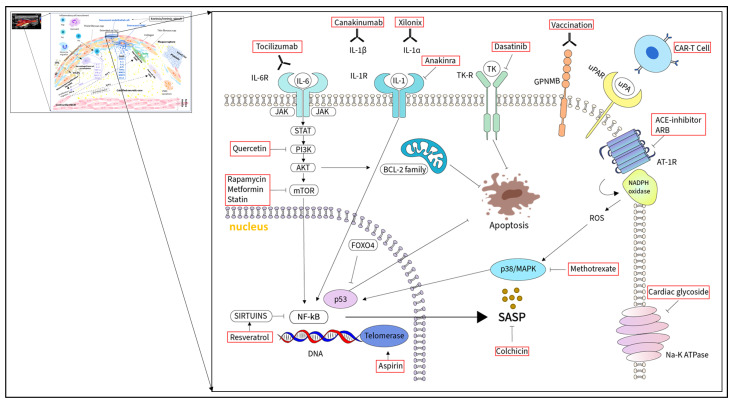
Schematic illustration highlighting targets of senotherapeutics including senolytics and senomorphics. Senolytics induce apoptosis in senescent cells by targeting key enzymes involved in anti-apoptotic and pro-survival mechanisms, such as tyrosine kinase (TK), p53, B-cell lymphoma-2 (Bcl-2) family proteins, serine/threonine protein kinase (AKT), phosphatidylinositol 3-kinase (PI3K) and forkhead box O protein (FOXO). Dasatinib is a TK inhibitor able to inhibit cell proliferation and induce apoptosis. (Figure 3) Quercetin is a flavonoid, which interacts with a PI3K isoform and Bcl-2 family members. Glycoprotein nonmetastatic melanoma protein B (GPNMB) and urokinase-type plasminogen activator receptor (uPAR) are seno-antigens broadly expressed on senescent cells. Peptide vaccination against GPNMB and CAR-T cells directed against uPAR can remove senescent cells. Senomorphics suppress SASP cell function by neutralizing SASP components or by interacting with their transcriptional regulators. Rapamycin inhibits the mammalian target of rapamycin (mTOR) signaling and decreases nuclear factor kappa B (NF-κB) activity to reduce interleukin 1α (IL-1α) production. Metformin and statins inhibit the mTOR signaling pathway. Aspirin increases nitric oxide synthesis and decreases oxidative stress, consequently up-regulates telomerase activity. Angiotensin-converting-enzyme inhibitors (ACEIs) and angiotensin II receptor blockers (ARBs, formally AT1R blockers) prevent telomeric and non-telomeric deoxyribonucleic acid (DNA) damage of angiotensin II through reactive oxygen species (ROS) mediated oxidative stress. Resveratrol activates sirtuins that regulate cellular senescence via targeting NF-κB transcription factors. Cytokine targeting therapy includes Xilonix, which is a monoclonal antibody specifically targeting IL-1α; canakinumab, which is a selective monoclonal antibody against interleukin 1β (IL-1β); anakinra, which is an IL-1 receptor antagonist; and tocilizumab, which is a monoclonal antibody against IL-6 receptor. Colchicine exerts broad anti-inflammatory effects, such as inhibition of IL-1 release. Methotrexate inhibits the p38/mitogen-activated protein kinase (p38/MAPK) pathway, however, it did not prove beneficial in patients with cardiovascular disease. NADPH oxidase: nicotinamide adenine dinucleotide oxidase, Na-K ATPase: sodium-potassium adenosine triphosphatase, JAK: Janus kinase, STAT: signal transducer and activator of transcription.

Atherosclerosis is an age-related cardiovascular disease and a major contributor to morbidity and mortality [1,2]. The public health burden of atherosclerosis is increasing due to the aging population and the growing prevalence of risk factors [3,4]. Data from the Global Burden of Disease revealed that life expectancy increased significantly in the last decades worldwide [3,4]. The World Population Prospects showed that the elderly (>65 years) represent nearly 15% of the world’s population [3,4]. Atherosclerosis is a low-progression, chronic inflammatory disease characterized by endothelial dysfunction, lipid infiltration, and foam cell generation leading to the formation of atherosclerotic plaque [5] (Figure 1 and Figure 2). Advancing age is a major risk factor for atherosclerosis, however, the interaction between aging, cardiovascular risk factors, and atherosclerosis is complex [6]. Aging is defined as the decline in cellular function over time as a consequence of several processes including genomic instability, telomere attrition, epigenetic alterations, loss of proteostasis, deregulated nutrient-sensing, mitochondrial dysfunction, cellular senescence, stem cell exhaustion, and altered intercellular communication [7]. Both aging and extracellular stress factors, such as mechanical and oxidative stress, can induce cellular senescence of vascular tissue and immune cells [8,9]. These senescent cells can accumulate and contribute to plaque initiation, formation, and plaque instability. Apart from cellular senescence, clonal hematopoiesis of indeterminate potential (CHIP) represents a novel age-related cardiovascular risk factor. This denotes a somatic mutation of hematopoietic stem cells, which leads to a competitive advantage of this cell clone expansion [10] (Figure 3). Anti-aging therapies and nutritional approaches have been studied to remove senescent cells (senolytics) or suppress senescent cell functions (senomorphics) in order to treat or prevent age-related diseases, such as atherosclerosis. The present review provides an overview of cellular alterations in atherosclerosis focusing on cellular senescence and a discussion of potential senotherapeutic strategies in this field. Aging research might help to understand the complex molecular and cellular processes in cellular senescence to develop novel preventive and therapeutic plans to reduce the health burden of atherosclerosis.

## 2. The Concept of Cellular Senescence

Cell senescence is implicated in numerous age-related diseases such as atherosclerosis. Cellular senescence is described as an irreversible cell cycle arrest state due to intrinsic (replicative cellular senescence) or extrinsic factors (stress-induced cellular senescence) [9,11]. The telomere is a nucleoprotein complex at the end of the chromosomes with tandem repeats of deoxyribonucleic acid (DNA). The replicative senescence is mainly explained by critical telomere shortening with each somatic cell division as the DNA polymerase is unable to fully replicate the chromosome ends. This results in DNA loss at each cell division and initiates a DNA damage response when a critical telomere shortening is reached with aging (intrinsic cellular stress) [9]. This activates the negative regulators of the cell cycle such as the p53–p21 and p16 tumor suppressor pathway [9,12]. Besides intrinsic cellular stress, a large number of extrinsic cellular stress factors can induce cellular senescence, defined as stress-induced premature cellular senescence [6,9]. These stressors include mechanical, oxidative, and metabolic stimuli leading to DNA damage and mitochondrial dysfunction [6,9,13]. Cardiovascular modifiable risk factors, such as hypertension, diabetes mellitus, hyperlipidemia, obesity, and smoking are the most common stressors inducing the formation of premature senescence of vascular cells [6,14,15]. Similar to replicative senescence, the p53/p21, and p16 anti-apoptotic and pro-survival pathways are upregulated in the premature senescent cells. These involve several key proteins, such as B-cell lymphoma-2 (BCL-2) protein family, phosphatidylinositol 3-kinase (PI3K), Janus kinase/ signal transducer and activator of transcription (JAK/STAT) protein, serine-threonine protein kinase (AKT), mammalian target of rapamycin (mTOR) protein, forkhead box O4 (FOXO4), nuclear factor-κB (NF-κB), tyrosine kinase, heat shock protein 90 (Hsp90) and silencing information regulator-related enzymes (SIRT, sirtuins) [12] (Figure 3). In the last two decades, sirtuins came into the forefront as a family of nicotinamide adenine dinucleotide (NAD)-dependent histone and non-histone deacetylases that regulate cellular senescence via targeting FOXO and NF-κB transcription factors [16] (Figure 3). In addition, the evolutionarily conserved p38/mitogen-activated protein kinase (p38/MAPK) pathway is also activated in premature senescent cells [17]. Importantly, senescent cells are not inactive cells, as they secrete a number of cytokines, chemokines, and matrix metalloproteinases defined as SASP [18]. This contributes to vascular structural and functional remodeling associated with aging and other cardiovascular risk factors [9] (Figure 3).

In summary, cellular senescence is a known major contributor to the development of atherosclerosis, which affects multiple cell types involved in atherosclerotic plaque, such as endothelial cells, vascular smooth muscle cells, and immune cells discussed below [13,19].

## 3. Normal Vessel Wall

The blood vessel wall consists of three layers: tunica intima (the inner layer), tunica media (the middle layer), and tunica adventitia (the outer layer). The tunica intima contains a single layer of endothelial cells (defined as endothelium) and a subendothelial layer (defined as basal lamina), which is made up of connective tissue. The endothelium provides a semi-permeable barrier between blood and vessel wall tissue. Furthermore, it responds to mechanical and hormonal signals, regulates adhesion and trans-endothelial migration of inflammatory cells, affects coagulation by expressing non-thrombogenic factors, and modifies vascular tone. The proliferative capacity of endothelial cells is limited with age [8]. The major cellular components of tunica media are the vascular smooth muscle cells (VSMC) surrounded by the extracellular matrix to provide elasticity of the blood vessel wall. Recent literature classifies six different VSMC and VSMC-like phenotypes, such as contractile, mesenchymal-like, fibroblast-like, macrophage-like, osteogenic-like, and adipocyte-like phenotypes. In healthy vessels, the contractile VSMC form is the most frequent cell type characterized by spindle-shape morphology and contractile marker expression [20,21,22]. In arteries with atherosclerotic plaques, the contractile VSMCs provide arterial wall remodeling to sustain blood flow. Furthermore, the contractile VSMC phenotype can switch to other VSMC-like phenotypes, which contributes to the formation of the atherosclerotic plaque and its vulnerability discussed in detail below [23].

## 4. The Complex Pathophysiology of Atherosclerosis

Atherosclerosis is a chronic inflammatory disease characterized by the complex interactions between endothelial cells, VSMCs, monocytes, macrophages, and lymphocytes in the vascular wall as a consequence of aging and non-aging-related pathways including cellular senescence [9,22,24]. Beyond the known “simplified” course of atherosclerosis, such as intima thickening, and stable and unstable plaque formation, the widespread application of new sensitive imaging tools allowed the differentiation of further atherosclerotic entities [25]. These new features include “superficial erosion” and “plaque healing”, highlighting the complex nature of the disease [25].

Extracellular stimuli, such as low shear stress, oxidative stress, and inflammatory cytokines promote premature stress-induced endothelial cell senescence leading to endothelial dysfunction. In addition, age-related replicative endothelial cell senescence further contributes to endothelial dysfunction. The senescent and damaged endothelial cells show decreased nitric oxide (NO) production, increased vascular permeability, and expression of surface adhesion molecules, such as intercellular adhesion molecule 1 (ICAM-1) and vascular cell adhesion protein 1 (VCAM-1) [26]. These promote the infiltration of inflammatory cells and lipids into the subendothelial space [26]. In the early stage, low-density lipoprotein (LDL) particles accumulate and undergo oxidative modifications in the subendothelial space becoming immunogenic (oxidized low-density lipoprotein, ox-LDL). In the subendothelial space, the monocytes differentiate into macrophages consuming lipids and forming foam cells. Foam cells release several inflammatory cytokines, growth factors, and chemokines, such as interleukin-1 (IL-1), interleukin-6 (IL-6), tumor necrosis factor-α (TNF-α), interferon-γ (IFN-γ), platelet-derived growth factor (PDGF) and monocyte chemokine protein-1 (MCP-1) [19]. In addition to monocytes, senescent vascular endothelial cells also secrete MCP-1 which recruits more peripheral monocytes to the endothelium and boosts their foam cell formation. The accumulation of foam cells leads to lipid core development. (Figure 2). The contractile VMSCs switch phenotype to mesenchymal-like and myofibroblast-like VMSCs, migrate and proliferate in response to PDGF, and synthesize extracellular matrices, such as collagen, elastin, and proteoglycans [5] (Figure 2). This leads to intima thickness in the early phase of atherosclerosis, and to the formation of fibrous cap in the advanced phase [5]. Furthermore, endothelial and macrophage cells that have undergone endothelial-to-mesenchymal transition or macrophage-to-mesenchymal transition also produce extracellular matrices [27]. The fibrous cap is composed mainly of a collagen-proteoglycan matrix and encloses the growing lipid core from the blood flow [22]. Calcification is initiated as microcalcifications, which are defined as small calcium deposits (<5 µm) due to cell death within the lipid core [28,29]. Lymphocytes also invade into the subendothelial space exerting dual roles in atherogenesis [5]. Activated T-helper 1 (TH1) lymphocytes promote atherosclerosis by stimulating mononuclear phagocytes via IFNγ. Oppositely, T-helper 2 (TH2) lymphocytes and regulatory T (Treg) cells produce the anti-inflammatory cytokine interleukin-10 (IL-10) and tumor growth factor β (TGFβ). Notably, platelets also contribute to plaque formation [30]. Flow-activated endothelial cells increase the expression of adhesion molecules interacting with the receptors of activated platelets [30]. These activated platelets recruit circulating monocytes by releasing inflammatory factors [30].

In the advanced course of atherosclerosis, apoptotic foam cells, and debris accumulate due to their impaired clearance; forming the necrotic, lipid-rich core of the atheroma [5]. Stable plaques have a thicker fibrous cap, which is enriched with collagen and cells undergoing mesenchymal transition. The necrotic core is smaller in the case of stable plaques compared to unstable plaques [22]. Extracellular matrix degradation and reduced collagen synthesis lead to fibrous cap thinning mainly due to both endothelial and VSMC-derived SASP senescent cells, macrophage-like VSMCs, and neutrophil cells releasing matrix metalloproteinase-9 (MMP-9) [22,24] (Figure 2). Unstable plaques with thin fibrous caps are prone to rupture, which triggers blood clot formation, defined as atherothrombosis, leading to arterial occlusion [22,24] (Figure 2).

## 5. Cells in Atherosclerosis: Focus on Cellular Senescence

### 5.1. Vascular Endothelial Cells

Emerging evidence suggests that senescent endothelial cells have a pivotal role in atherosclerosis [19,31]. Hemodynamic environment in blood vessels and cardiovascular risk factors can promote endothelial senescence mainly via mechanical and oxidative stress. It is known that the rate of endothelial cell turnover is higher at sites of branches and curvatures with low flow and oscillatory shear stress compared to laminar flow [9,32]. (Figure 1) Endothelial cell turnover occurs mainly via mitotic endothelial cell division rather than replacement by progenitor cells [33]. The higher division rate at sites of branches and curvatures leads to critical telomere attrition and telomere uncapping, which initiates a DNA damage response and activates the p53–p21 pathway resulting in replicative senescence [9,33,34]. Furthermore, oxidative stress is higher at the sites of arterial branches with higher mechanical stress compared to laminar blood flow sites [9] (Figure 1). Hypertension and the consequent elevated pulse pressure represent further mechanical stress triggering premature stress-induced endothelial cell senescence. This reduces NO levels and increases angiotensin II and endothelin I levels creating a vicious circle between blood pressure elevation and senescence aggravation [9,19,35]. Furthermore, senescent endothelial cells promote pathways of arterial remodeling leading to arterial stiffening and hypertensive disease, which in turn initiates the progression of senescence and atherosclerosis [36]. Obesity, smoking, hyperlipidemia, and diabetes mellitus represent further cardiovascular risk factors promoting premature endothelial cell senescence and atherosclerosis [9,37,38]. Notably, these cardiovascular risk factors are partially age-related factors, showing the pathological complexity between age, cellular senescence, and atherosclerosis. Hyperlipidemia leads to increased oxidation of low-density-lipoproteins, which activates the PI3K/AKT1 signaling pathway and increases the level of ROS [19]. Diabetes mellitus and high serum glucose also promote ROS level elevation in the same way. In addition, high serum glucose level induces NADPH oxidase-, superoxide dismutase- (SOD), and sirtuin-mediated ROS accumulation [19,37,39]. Notably, ROS can also trigger the DNA damage response pathway, which induces endothelial senescence [19]. SIRT1 is one of the most studied sirtuins from the family, which targets histones and non-histone proteins (such as FOXO, p53, NF-κB transcription factors) and regulates DNA damage repair [12,40,41]. Previous studies showed, that SIRT1 attenuates oxidative stress, inhibits endothelial cell activation, prevents foam cell formation, activates endothelial nitric oxide synthase (eNOS), and promotes NO production [16,42,43,44]. Consequently, SIRT1 can protect against endothelial senescence and atherosclerosis.

Senescent endothelial cells show morphological and functional changes compared to normal endothelial cells leading to endothelial dysfunction. The adhesion of the enlarged senescence endothelial cells to the basal membrane is increased leading to impaired alignment to laminar shear stress, but resistance to denudation [45]. Aging results in the accumulation of these cells owing to the imbalance between endothelial senescent cell production and elimination, due to age-related immunosenescence [46]. Endothelial senescent cell accumulation leads to endothelial barrier dysfunction, increased levels of inflammatory cytokines (IL-1β, IL-6, IL-8), chemokines (chemokine ligand 11, MCP-1), growth factors, and ROS. These processes facilitate immune cell infiltration, meanwhile, the vasodilatory NO production decreases [9,19,47]. Importantly, senescent endothelial cells express high levels of plasminogen activator-1 (PAI-1) and low levels of eNOS, which increases the thrombosis risk and susceptibility to atherosclerosis [18,19].

In addition, senescence induction and extracellular stimuli can stimulate endothelial cell phenotype switch; however, the activation pathway is still scarcely understood. The endothelial-to-mesenchymal transition (EndoMT) is a process defined as a change in cell morphology, loss of eNOS and endothelial markers, expression of mesenchymal and smooth muscle cell markers, and production of extracellular matrix and pro-inflammatory proteins [48].

### 5.2. Vascular Smooth Muscle Cells

Growing evidence suggests that replicative senescent VSMCs accumulate with aging and premature stress-induced senescent VSMCs are induced by chronic inflammation and oxidative stress. In both cases, the senescent VSMCs show SASP-VSMCs and secrete inflammatory cytokines such as IL-1, IL-6, IL-8, C-C motif chemokine ligand 2 (CCL2), MCP1, and macrophage inflammatory protein-1α/β and CCL3/4 [22,49]. These cytokines promote the recruitment of immune cells, thereby accelerating plaque growth and rupture [13]. Cardiovascular risk factors, such as aging, diabetes, hypertension, dyslipidemia, and smoking increase the level of inflammatory cytokines, and the level of ROS, which promote VSMC senescence [19,50]. The Renin-Angiotensin-Aldosterone System (RAAS) activation, the chronic exposure to high levels of the coagulation factor Xa, and the MCP-1 and TGF-ß signaling pathways are further VSMC senescence inducers [19,51,52]. Furthermore, senescent VSMCs secrete a large amount of MMPs for decomposing elastin into fragments resulting in vascular stiffness increase. In addition, SASP-VSMCs overexpress integrin proteins, focal adhesion proteins, and cytoskeleton proteins, therefore, the interaction between extracellular matrix (ECM) and VSMCs increases leading to further vascular stiffness increase [22]. Nevertheless, the production of collagen by senescent VSMCs is reduced, promoting atherosclerotic plaque vulnerability. Notably, when the vessel wall is damaged due to extrinsic or intrinsic cellular stimuli, the contractile VSMCs can turn to senescence or switch their phenotype to a synthetic VSMC-like phenotype as a response to vascular damage [22,23]. Importantly, the secretory VSMC-like cells can migrate and proliferate, which separates them from SASP-VSMCs showing irreversible cell cycle arrest [22].

Previous studies confirmed a substantial heterogeneity among VSMC phenotypes within the aged arterial wall suggesting that both senescent and proliferative VSMC subsets may coexist. The phenotypic transition of contractile VMSC to other secretory VMSC-like phenotypes leads to the loss of VSMC-specific markers [23]. Recently, multiple signaling pathways have been identified that regulate the VSMC phenotypic switch under the induction of extrinsic stimuli [23]. The dedifferentiation of contractile VSMC phenotype to mesenchymal-like phenotype is induced by the KLF4, which is one of the Yamanaka transcription factors [20]. This phenotype switch is modulated by platelet-derived growth factor (PDGF-BB) and retinoic acid [53,54,55]. The mesenchymal-like VSMC might further shift to myofibroblast-like VSMC, which is regulated by transcription factor 21 (TCF21) and considered a protective phenotype in atherosclerosis [56]. In the initiation phase of atherosclerosis, ox-LDL, and the inflammatory cytokines stimulate the contractile VSMC to myofibroblast-like VSMC transition and the migration of these cells from tunica media to tunica intima. These cells exhibit reduced levels of contractile markers and show many features of fibroblast-derived myofibroblasts, such as proliferation, migration, and collagen deposition forming the fibrous cap [57]. Furthermore, myofibroblast-like VSMCs can transform into macrophage-like VSMCs, osteogenic VSMCs, and other types of VSMCs [20]. High intracellular cholesterol exposure induces KLF4 expression in VSMC, which promotes foam cell-like VSMC formation regardless of its cholesterol unloading capacity [58,59]. Cholesterol and oxLDL activate genes that regulate cholesterol efflux from VSMC, such as ATP-binding cassette transporter 1 (*ABCA1*) and ATP-binding cassette transporter G1 (*ABCG1*) [60]. Macrophage-like VSMCs secrete MMPs, which destroy ECM in the fibrous cap and induce plaque vulnerability [22]. Furthermore, KLF4 might enhance osteogenic-like VSMC differentiation from mesenchymal-like VSMC phenotype via regulation of Runt-related transcription factor 2 (RUNX2) and SRY-Box Transcription Factor 9 (SOX9) [20,61]. These cells lead to the deposition of calcium in the intimal layer resulting in calcification and obstruction [20,61,62]. Contractile to adipocyte-like VMSC switch is the least studied VMSC phenotype conversion [20]. KLF10 (also defined as a TGF-inducible early gene) is supposed to regulate adipocyte-like VMSC transition [63].

### 5.3. Immune Cells

Chronic, low-grade, “sterile” arterial inflammation is the main contributor to the development of diffuse intimal-medial thickening, disruption of the endothelium, VSMC migration, VSMC senescence, extracellular matrix deposition, elastin fracture, and matrix calcification. Macrophages and senescent cells are the principal sources of pro-inflammatory cytokines such as IL-1β, IL-6, and TNF during aging [64]. The dominant immune cells within atherosclerotic plaques are macrophages, which play a critical role in plaque initiation and progression via inflammation, cholesterol efflux, and ECM degradation [65]. In addition to macrophages, monocytes, and lymphocytes also have a significant role in atherosclerosis. The implementation of lymphocytes in atherosclerosis is dual: T-helper 1 and B2 lymphocytes promote atherosclerosis, however, T-helper 2, regulatory T cells, and B1 lymphocytes can mute this process [24]. Immunosenescence is one of the mechanisms that contribute to the onset of chronic, low-grade inflammation. This is because immune cells can also undergo cellular senescence and accumulate with age, nonetheless, the accumulation of any monocyte subtype is debatable [66,67,68]. Immune cells eliminate senescent cells in general, however, senescent immune cells are unable to perform this function. This results in the accumulation of senescent endothelial cells, VSMCs, macrophages, dendritic cells, and foam cells. Pro-inflammatory mediators released from these cells aggravate atherosclerotic plaque formation. Notably, senescent immune cells may also contribute to plaque reduction, because senescent lymphocytes fail activation when stimulated by macrophages and dendritic cells. In addition, senescent macrophages decline phagocytosis, antigen presentation, and cytokine production [65,69].

#### 5.3.1. Monocytes

The monocyte population is heterogeneous compromising three monocyte subsets: the classical (CD14++CD16−), the intermediate (CD14++CD16+), and the non-classical (CD14+CD16++) monocyte subpopulations [65,68]. Ong and coworkers found that the non-classical subset shows the highest level of senescence, followed by the intermediate and then the classical subset [68]. The working group demonstrated, that non-classical monocytes show the most characteristics of SASP. They are at least proliferative population, have the shortest telomere length, have high concentrations of cellular and mitochondrial ROS, and exhibit the highest levels of phosphorylated transcription factor NF-κB [68]. In conclusion, a progressive transition was presumed toward senescence from the classical to the intermediate and to the non-classical subset [68]. Senescent monocytes have increased phagocytic activity, express pro-atherogenic chemokine receptors and endothelial adhesion molecules, such as VCAM-1 and ICAM-1, and show TNF-α and IL-1β secretion. These result in a consequent increased endothelial adhesion capacity of monocytes [68].

#### 5.3.2. Macrophages

Macrophages have a key role in the pathogenesis of atherosclerosis, including oxLDL uptake, lipid accumulation, foam cell formation, and pro- and anti-inflammatory response. It is widely accepted that the macrophage population is heterogeneous [70,71,72,73]. Cochain and coworkers revealed three subtypes of macrophages in the atherosclerotic plaque using differential gene expression and gene ontology enrichment analyses [73]. The three subtypes include resident-like macrophages, inflammatory macrophages, and TREM2^hi^ (triggered receptor expressed on myeloid cells 2) macrophages [73]. Although, inflammatory and resident-like macrophages might resemble the traditionally classified M1- (pro-inflammatory) and M2- (alternative, anti-inflammatory) polarized macrophages, Cochain and coworkers found a significant overlap in the expression of markers used to differentiate the M1- and M2-macrophages. The TREM2^hi^ macrophages are osteoclast-like phenotypes, which have a key role in atherosclerotic lesion calcification. Whether vascular TREM2^hi^ macrophages differentiate locally from vascular resident macrophages or other sources remains debatable [73]. Lin and coworkers assumed that the previous association of M1 macrophages with atherosclerotic plaque progression and M2 macrophages with plaque regression might be a rough simplification of a complex network [74]. The number of M2-macrophages was higher during plaque regression, however, a cluster of M2 macrophages was present in plaque progression [74]. Nevertheless, all three subtypes of macrophages in the atherosclerotic plaque can show senescence features, including resident-like macrophages, inflammatory macrophages, and TREM2^hi^ macrophages [74]. Secretion of SASPs (IL-6, TNF, and chemokines), expression of senescence markers (CXCL4, CCR2, and CXC3R1), expression of ligands that enhance antigen presentation and T-cell recruitment (CD 80) and high expression of CD9 glycoprotein that activates (PI3K)/AKT/mTOR signaling pathway were associated with the main senescence features of macrophages [75]. In addition, senescent macrophages have low expression of toll-like receptors (TLRs) for antigen presentation to the effector T-cells to initiate inflammatory responses and have impaired cholesterol efflux due to the downregulation of *ABCA1* and *ABCG1* genes [65,76].

#### 5.3.3. Dendritic Cells

Dendritic cells are key antigen-presenting cell subsets involved in inflammation and thought to instruct lymphocytes, produce MMPs and collagenases to destabilize atherosclerotic plaque, secrete IFN-α, IFN-β, and CCL-5 to promote the maturation of further dendritic cells [65,77,78,79]. Furthermore, dendritic cells can take up oxLDL by exophagy and can transform into foam cells [65,77,78,79]. They are classified according to the expression of markers into conventional, plasmacytoid, and monocyte-derived dendritic cells [77]. Conventional dendritic cells promote T-helper 2, T-helper 17, or Treg responses [77,80]. Plasmacytoid dendritic cells are activated by immune complexes containing self-DNA from dying cells in the plaque and enhance the immune response against self-molecules thus promoting atherosclerosis progression [81]. Monocyte-derived dendritic cells engulf lipids and become foam cells similar to macrophages [82]. The number of dendritic cell numbers in atherosclerotic plaques positively correlates with plaque vulnerability [83]. However, previous studies using mouse models revealed both pro-atherogenic (CCL17+) and anti-atherogenic (CD103+) functions of dendritic cells [84,85,86]. Aging could affect the activation and maturation of dendritic cells, which can subsequently become reactive to circulating self-antigens from apoptotic cells secreting IFN-α and IL-6 [87,88]. Agrawal and coworkers observed increased basal levels of NF-κB activation in dendritic cells from aged subjects [88]. Overall, dendritic cells from aged subjects are characterized by the generation of immune responses to self-antigens and loss of tolerance [89].

#### 5.3.4. Neutrophil Cells

Neutrophil cells have a pro-atherogenic role via secreting chemotactic molecules and reactive oxygen species promoting inflammation and increased endothelial cell permeability [10]. Furthermore, neutrophil cells can extrude their nuclear material, defined as neutrophil extracellular traps (NETs), which can bind to VSMCs and induce cell lysis and plaque destabilization [10,90,91,92]. Data on the possible involvement of aging neutrophils in atherogenesis are limited [93]. Aged neutrophils change phenotype, form NETs, produce ROS, and consequently are involved in the development and progression of atherosclerosis [93].

#### 5.3.5. T Cells

T cells represent the largest population of leukocytes in the atherosclerotic plaque with a large phenotype diversity and promote both initiation and progression of atherosclerosis [94]. Besides their pivotal pro-atherogenic role, T cells have been shown to have an anti-atherogenic role as well, depending on the T cell subset. Three main lesional T cell subgroups have been separated: CD4+, non-conventional, and CD8+ T cells [95]. The CD4+ T cell subgroup can be divided into further three subsets, such as CD4+ T helper 1, CD4+ T helper 2, and CD4+ T regulatory cells [95]. The CD4+ T helper1 subset can express IFN-γ and is thought to promote atherogenesis [95]. CD4+ T helper 2 cells express IL-4 and IL-13, but their impact on atherosclerosis is controversial mainly due to the dubious function of IL-4 [96,97]. Furthermore, CD4+ T regulatory cells are supposed to have an atheroprotective role via IL-10 and TGFβ secretion, however, in the late stage of atherosclerosis, they can convert into pro-atherogenic ex-T regulatory cells [98]. Non-conventional T cells include the pro-atherogenic natural killer T (iNKT) cells and the IL-17 expressing γδ T cells. iNKT cells are supposed to influence the initial phase and advanced stages of atherosclerosis by plaque destabilization [99]. CD8+ T cells have been also described to exhibit pro-atherosclerotic properties via IFNγ production and macrophage activation, and anti-atherosclerotic properties via B cell modulation; however, knowledge of their role in atherosclerosis is limited [95].

Senescent T-cells show over-activation of the p38/MAPK signaling pathway, which triggers IFN-γ expression and thereby promotes atherosclerotic plaque development [100]. Senescent CD4+ T-cells highly secrete CCR5, CCR7, and CXCR1 chemokine receptors, which promote inflammation at the site of atherosclerotic plaque [65]. T-cell numbers decline with aging due to the accumulation of cholesterol in the aging T-cells, which may induce T-cell apoptosis or T-cell exhaustion [101,102,103]. The majority of CD8+CD28+ T cells in human peripheral blood will become progressively differentiated and lose their CD28 antigen expression in the course of immunosenescence. Sirtuin-1 is down-regulated in terminally differentiated CD8+CD28− memory T cells which leads to decreased FOXO1 expression [104]. FOXO1 enhances the release of pro-inflammatory cytokines and controls macrophage oxLDL uptake and vascular calcification, consequently, senescent CD8+ CD28− T cells might slow atherosclerosis via SIRT1/FOXO1 decline [65].

#### 5.3.6. B Cells

B cells are usually localized in the adventitia and not in atherosclerotic plaques. B cells are classified into atheroprotective B1 and pro-atherogenic B2 cells [105]. The level of senescent late memory B-cell in the peripheral blood of healthy elderly individuals is elevated [106]. These cells express high levels of SASP biomarkers, such as TNF-α, IL-6, IL-8, and pro-inflammatory miRNAs [65,107]. MiRNA-155 accelerates foam cell formation, down-regulates endothelial NO synthase, and reduces oxLDL uptake [108]. Late memory B cells express higher levels of CXCR3 with aging, which enables the homing of these cells to the sites of inflammation [106]. Interestingly, inflamed endothelial cells express higher levels of CXCR3 ligand, therefore, B cells can directly enter vascular tissue from the blood [106,109]. The energy-sensing enzyme AMP-activated protein kinase (AMPK) is activated in senescent late memory B cells leading to NF-kB activation [106,110].

### 5.4. Hematopoietic Stem Cells and Progenitor Cells

Somatic mutation in a hematopoietic stem cell results in mutation transfer into the fraction of its derived cells. Clonal hematopoiesis refers to the proliferation of stem cells in response to a mutation, which is common in aging and is strongly associated with atherosclerosis [111,112,113,114]. The risk of mutation is higher during stem cell proliferation, consequently, increased hematopoiesis promotes mutations [115]. Mutations of loss-of-function methylcytosine dioxygenase 2 (*TET2*), DNA (cytosine-5)-methyltransferase 3A (*DNTM3A*), polycomb chromatin-binding protein (*ASXL1*), and gain-of-function *JAK2* transcriptional regulator genes result in positive selection and expansion of clones of hematopoietic cells without malignancy. This phenomenon is defined as CHIP [113,116]. Previous studies showed, that *TET2* or *JAK2* clonal mutation increases IL-6 and IL-1β production in myeloid cells supporting accelerated atherosclerosis [112,113,114]. These studies suggested a link between atherosclerosis and hematopoiesis. Overall, age-related CHIP has been recognized as a risk factor for atherosclerosis [10,113].

In case of endothelial injury (e.g., due to mechanical stress), endothelial repair might be mediated either by endothelial progenitor cells (EPCs) or via mitotic cell division from injury-adjacent endothelial cells [9,33,117,118]. Endothelial progenitor cells are mobilized from the bone marrow to the circulation due to substances secreted during vascular injury (e.g., growth factors). Later EPCs migrate toward damaged endothelial regions where they adhere, further proliferate, differentiate, and secrete cytokines and growth factors enhancing the replication of mature endothelial cells [119,120]. Cardiovascular risk factors can influence the above-mentioned EPC performance contributing to endothelial dysfunction [119,121]. EPC senescence can contribute to numerical and functional impairments of EPCs with aging and the presence of cardiovascular risk factors [122,123].

## 6. Senotherapeutics in Atherosclerosis

Senotherapeutics involve mainly senolytics and senomorphics [18]. Senolytics induce apoptosis in senescent cells by targeting key enzymes involved in anti-apoptotic and pro-survival mechanisms, such as tyrosine kinase, p53, p21, Bcl-2 family proteins, AKT, PI3K, and FOXO4. In contrast, senomorphics suppress the effects of SASPs without causing cell death, and target NF-κB, mTOR, IL-1, p38/MAPK, and other signaling pathways [17,18]. (Figure 3) In addition, immunotherapies targeting cytokines might be also defined as senomorphics, as cytokines can be produced either by senescent or non-senescent cells. There is growing interest in developing senotherapeutics in the field of atherosclerosis due to the promising results of clinical and preclinical trials. However, the distinction between beneficial and pathological senescence is essential and should be taken into account in future clinical trials.

### 6.1. Senolytics

The first reported senolytics were dasatinib and quercetin. Dasatinib is a tyrosine kinase inhibitor able to inhibit cell proliferation and induce apoptosis. In Figure 3, quercetin is an anti-oxidant flavonoid found in fruits and vegetables, which interacts with a PI3K isoform and Bcl-2 family members [17]. In Figure 3, it is established that coronary artery bypass graft (CABG) surgery triggers a perioperative inflammatory response which may contribute to the genesis of common postoperative complications [124]. The Q-CABG (NCT04907253) is an ongoing phase II, single-center, prospective, randomized, double-blind, placebo-controlled study evaluating the anti-inflammatory and senolytic effects of quercetin vs. placebo in patients undergoing CABG [124]. Further senolytics, such as inhibitors of the anti-apoptotic BCL-2 family proteins (e.g., navitoclax), heat shock protein 90 inhibitors, ubiquitin-specific peptidase 7 (USP7) inhibitors, p53 modulators (e.g., inhibitors of FOXO4-p53), Na+/K+-ATPase inhibitors (e.g., cardiac glycosides), and others have been used so far in phase I and II clinical trials. However, these trials evaluated the effect of these drugs only in osteoarthritis, macular degeneration, idiopathic pulmonary disease, Alzheimer’s disease, and diabetic kidney disease [17]. Notably, most senolytic agents non-selectively inhibit the anti-apoptotic pathways, consequently, it might raise concerns about efficacy and off-target unwanted side effects in normal tissues [125]. Nevertheless, substantial progress has been achieved in developing anti-senescent cell antibodies and vaccines [19,126]. Glycoprotein nonmetastatic melanoma protein B (GPNMB) is expressed at high levels in senescent cells and is defined as a senescence-specific protein (seno-antigen) [125]. Suda and coworkers demonstrated that GPNMB expression is upregulated in the case of atherosclerosis in vascular endothelial cells and leukocytes [125]. Furthermore, peptide vaccination against GPNMB eliminated GPNMB-positive senescent cells, reducing atherosclerotic plaque burden and metabolic dysfunction such as glucose intolerance in mouse models of obesity and atherosclerosis [125]. Immunotherapies developed in the field of oncology, such as chimeric antigen receptor T (CAR-T) cells, could be repurposed as senolytics in atherosclerosis by targeting seno-antigens with the advantage of reducing off-target effects [19] (Figure 3). Urokinase-type plasminogen activator receptors are broadly expressed on senescent cells in atherosclerotic plaques and might be used as seno-antigens [19,126]. Amor and coworkers demonstrated that CAR-T cells directed against urokinase-type plasminogen activator receptors removed senescent cells and improved glucose metabolism [127]. Senescent T cells, defined as CD4+ CD44^high^ CD62L^low^ PD-1+ CD153+ cells, accumulate in visceral adipose tissues triggering chronic inflammation, metabolic disorders, and cardiovascular diseases [128]. Yoshida and coworkers found that a vaccine targeting CD153-expressing senescent T cells can prevent the accumulation of senescent T-cells and improve glucose metabolism [129].

### 6.2. Senomorphics

Senomorphics suppress SASP cell function by neutralizing SASP components or by interacting with transcriptional regulators of the SASP, such as inhibitors of ataxia-telangiectasia mutated (ATM) serine-threonine kinase, p38/MAPK, JAK/STAT, and the NF-κB and mTOR pathways [17] (Figure 3). Rapamycin, metformin, and aspirin were among the first discovered senomorphic drugs [17] (Figure 3). Rapamycin (sirolimus) inhibits mTOR signaling and decreases NF-κB activity to reduce IL-1α production [17,130]. The senomorphic and anti-inflammatory effect of rapamycin was shown in senescent pulmonary vascular endothelial cells and animal models [131,132]. Lesniewski and coworkers demonstrated arterial function improvement in old mice treated with rapamycin. This was associated with reduced oxidative stress, 5′ AMPK activation, and increased expression of proteins involved in the control of the cell cycle [132]. These studies suggest that dietary rapamycin might counteract mechanisms responsible for arterial aging. However, it is mainly used as a local anti-proliferative agent in clinical practice. Sirolimus drug eluting coronary artery stents were introduced in clinical practice in 2002 to prevent injury-mediated intima hyperplasia leading to in-stent restenosis [133,134,135].

Metformin is a biguanide drug approved for the treatment of type 2 diabetes mellitus. In addition, the pleiotropic senomorphic effects of metformin have been revealed expanding its therapeutic potential through multiple pathways in cardiovascular and cancer diseases [17,136]. Metformin decreases the expression of angiotensin II receptor type I (AT1R) in the aortas of mice and attenuates vascular senescence and atherosclerosis [137]. Furthermore, metformin attenuates the high-fat diet-induced atherosclerosis, as it can diminish vascular senescence through its effect on the activation of AMPK and it can inhibit the mTOR signaling pathway [137,138]. Aspirin is a non-steroidal anti-inflammatory drug, which can inhibit senescence and SASPs by increasing nitric oxide synthesis and decreasing oxidative stress, consequently up-regulating telomerase activity and delaying senescence in endothelial cells [138,139]. It is known that statins, beyond lowering cholesterol levels, inhibit oxidative stress-induced endothelial senescence by up-regulating endothelial nitric oxide synthase, SIRT1, and inhibit the mTOR signaling pathway [140]. Emerging data support the beneficial pleiotropic cardiovascular effects of ACEIs and angiotensin II receptor blockers (ARBs, formally AT1R blockers) by preventing the telomeric and non-telomeric DNA damage of angiotensin II through ROS mediated oxidative stress [6,141]. Furthermore, ACEIs prevent endothelial cell senescence via bradykinin receptor-mediated inhibition of DNA damage [6,142]. Despite inhibiting senescence, Aspirin, metformin, statins, and ACEIs are not used as anti-aging senomorphic agents in the general population due to their side effects. These drugs are recommended for the treatment of cardiovascular risk factors [143].

Nutritional senomorphic and antioxidant substances found in plants might represent an option for primary atherosclerosis prevention with fewer side effects. The potential benefits of nutrient supplementations have been extensively investigated throughout recent decades in terms of anti-aging and anti-atherogenic effects due to their antioxidant and anti-inflammatory properties [144]. Growing evidence suggests that some natural phytochemicals, such as quercetin, resveratrol, green tea extract, curcumin, epicatechin, and cyanidin, can exert anti-aging effects by reducing oxidative stress, suppressing low-grade chronic inflammation, and inducing autophagy [145]. Resveratrol is the most studied phytochemical found in many plants, such as grapes, berries, cocoa, tomatoes, and peanuts [146]. The anti-oxidative and anti-inflammatory effects of resveratrol are only partly attributed to its structure containing free hydroxyl groups that can donate hydrogen atoms to protect cells against lipid peroxidation. It is known that this direct ROS scavenging effect of resveratrol is relatively poor [147,148]. The main in vivo antioxidant effect of resveratrol is more likely to be attributable to its gene regulation via SIRT1 leading to reduced ROS production from NADPH oxidases, and uncoupled eNOS [147]. In addition, resveratrol can upregulate anti-oxidative enzymes (such as superoxide dismutase, catalase, and NADPH quinone oxidoreductase 1) via SIRT1 and Nrf2 [44,145,148,149,150]. Ji and coworkers found that resveratrol downregulates the PI3K/AKT/mTOR signaling pathway in vitro in umbilical vein endothelial cells obtained from an atherosclerosis mice model [151]. It is known that mTOR plays a crucial role in regulating autophagy. Targeting the mTOR signaling pathway might exert a protective role against atherosclerosis, however, it is less known whether resveratrol can activate autophagy in vivo [44]. Furthermore, resveratrol inhibits the expression of ICAM-1 on endothelial cells, thereby blocking monocyte adhesion [152]. Notably, resveratrol has been shown to suppress VSMC migration, proliferation, and mineralization [146]. Nonetheless, the effect of resveratrol on different VSMC phenotype differentiation is debated [146,153]. Overall, resveratrol appears to have a significant therapeutic potential against aging and atherosclerosis, however, the number of clinical trials is limited and most of them use resveratrol in combination with other agents [148,154]. Agarwal and coworkers, in a double-blind, randomized, placebo-controlled clinical trial, evaluated the effects of a one-month combination treatment of 400 mg trans-resveratrol, 400 mg grape skin extract, and 100 mg quercetin on endothelial response and plasma biomarkers in 44 healthy individuals [155]. At the end of the treatment, an inverse relationship was observed between the concentration of plasma resveratrol and the expression of IL-8, VCAM, and ICAM. Furthermore, when the blood plasma of participants was incubated with cultured human coronary artery endothelial cells, the messenger RNA expression of IL-8, VCAM, and ICAM was reduced due to resveratrol treatment [155]. This study indicates, that resveratrol may have protective effects against atherosclerosis and might be considered as a primary preventive agent [155]. However, the preclinical and clinical studies of resveratrol in atherosclerosis and ischemic heart disease are inconsistent [146]. Importantly, the effects of resveratrol are dose-dependent, consequently, the results from studies using high doses of resveratrol are not representative of the effects of resveratrol when administered as a nutraceutical [146]. In addition, the bioavailability of resveratrol is poor due to its rapid metabolism, which represents a further challenge [144,146]. Nonetheless, the 2021 European Society of Cardiology Guidelines on cardiovascular disease prevention recommend choosing a more plant-based food pattern, rich in fiber, that includes whole grains, fruits, vegetables, pulses, and nuts [143]. Nuts are rich in bioactive and antioxidant compounds, such as polyphenols, tocopherols, phytosterols, and phenolics [143,156]. Previous studies have shown that nuts have beneficial effects on inflammation, lipid concentration, and endothelial function [157,158]. Furthermore, a meta-analysis from 18 prospective studies revealed significant inverse associations between frequent nut intake and cardiovascular disease supporting recommendations to include nuts as part of a healthy diet [156]. In the PREvención con DIeta MEDiterránea (PREDIMED) trial, Mediterranean diets supplemented with daily consumption of 30 g mixed nuts significantly reduced the risk of cardiovascular diseases and all-cause mortality compared with a low-fat diet [159].

Over the past years, promising results have been reported from clinical trials targeting inflammation in cardiovascular diseases. Several cytokine blockers have shown favorable results in atherosclerosis, including IL-1 and IL-6 blockade [10] (Figure 3). Both IL-1α and IL-1β are involved in atherosclerosis [10,160]. Vromman and coworkers demonstrated a crucial role for IL-1α in the remodeling of arteries during early atherogenesis, whereas IL-1β promotes mainly the growth of an established atheroma [160]. Xilonix is a monoclonal antibody specifically targeting IL-1α, canakinumab is a selective monoclonal antibody against IL-1β, and anakinra is an IL-1 receptor antagonist, which targets both IL-1α and IL-1β [10]. In Figure 3, a randomized, placebo-controlled phase II clinical trial with xilonix in patients undergoing percutaneous superficial femoral artery revascularization found no significant difference regarding restenosis between xilonix and placebo group at 12 months of follow-up [161]. The Canakinumab Anti-inflammatory Thrombosis Outcome Study (CANTOS) trial was a double-blind, randomized, controlled trial investigating the effects of canakinumab in patients with recent myocardial infarction [10]. The rate of cardiovascular events was significantly lower in the canakinumab group compared to the placebo group, which was independent of lipid-level lowering therapy [162]. However, there was no significant difference in all-cause mortality due to higher infection risk in the canakinumab group [162]. Previous randomized double-blind, placebo-controlled phase II clinical trials with anakinra (IL-1 receptor antagonist) showed significant hsCRP levels in patients with the acute coronary syndrome in the anakinra group compared with the placebo group [163,164]. Apart from IL-1, IL-6 represents a further crucial pro-inflammatory cytokine contributing to atherosclerosis and plaque destabilization. Previous phase II clinical trials with a monoclonal antibody against IL-6 receptor (tocilizumab) in myocardial infarction showed a significant hsCRP level decrease compared to the placebo group [165,166]. Despite the promising results of cytokine blockers in the field of cardiology, concerns have emerged about their infectious side effects suggesting the need for a more in-depth investigation regarding the optimal application of these drugs. This includes optimal patient selection, optimal cytokine blocker selection, and optimal timing of the treatment.

Colchicine exerts broad anti-inflammatory effects by inhibiting cytoskeletal microtubule formation, IL-1 release, and leukocyte motility [10] (Figure 3). Originally, it was used to treat pericarditis and gout [10,167]. In the Low-Dose Colchicine trials (LoDoCo and LoDoCo2), patients with stable chronic coronary artery disease and receiving colchicine had a 31% reduction in the incidence of the primary composite endpoint of cardiovascular death, myocardial infarction, ischemic stroke, and ischemic-driven coronary revascularization compared with patients receiving placebo [10,168,169]. The Colchicine Cardiovascular Outcomes (COLCOT) trial showed similar results in patients with myocardial infarction, supporting the beneficial effect of anti-inflammatory therapy in acute coronary disease [170]. Despite the promising milestone trials of CANTOS and LoDoCo, these results have not yet changed the treatment strategy in cardiovascular clinical practice [10]. Hydroxychloroquine is another broad anti-inflammatory drug that reduces the production of pro-inflammatory cytokines. Originally, this drug was used in malaria and inflammatory rheumatic diseases. However, currently, it is being tested in cardiovascular trials due to the observed decrease in cardiovascular events in rheumatic arthritis and systemic lupus erythematosus treated with hydroxychloroquine [10,171].

There are plenty of preclinical trials searching for further treatment strategies in atherosclerosis, such as JAK2 inhibitors and chemokine receptor targeting therapies [172,173,174]. Previous animal models showed that JAK2 inhibitors attenuate atherosclerosis in mice and rabbit models [172,173]. Chemokine receptor targeting therapies reduced atherosclerosis in mice by inhibiting monocyte/macrophage recruitment [174]. However, there are some treatment approaches in atherogenesis, such as TNF blockers, IL-23 blockers, and p38/MAPK inhibitors (such as methotrexate) that did not prove beneficial in patients with cardiovascular disease [10,175,176].

## 7. Conclusions

The interplay between vascular endothelial cells, vascular smooth muscle cells, immune cells, senescent cells, and lipid accumulation is pivotal in atherosclerosis. Cellular senescence is a substantial contributor to atherosclerosis including senescence of endothelial cells, vascular smooth muscle cells, and several immune cell types [177]. Senescence can be induced either by aging (replicative senescence) or/and oxidative and mechanical stress (premature senescence). Senescent cells exert detrimental effects through cellular dysfunction and via secreting a variety of inflammatory cytokines, immune modulators, and proteases, defined as senescence-associated secretory phenotypes. Senescent cells contribute to the degeneration and thinning of the atherosclerotic fibrous cap [178]. However, senescent cells also play a beneficial role in some cellular processes such as tissue repair and wound healing, therefore, the distinction between beneficial and pathological senescence is essential. In the last decade, substantial progress has been made in the understanding of cellular senescence, including its pathophysiology and senotherapeutical potentials. There is a growing interest in developing senotherapeutics targeting senescent cells in atherosclerosis, particularly in the field of immunotherapies due to the promising results of previous clinical trials [17,18]. Notably, stress factors can induce not only senescence but also non-senescence associated pathological cellular changes, both leading to the complex process of atherosclerosis. This includes chronic inflammation with the secretion of cytokines, proteases, immune modulators, and reactive oxygen species from both senescent and non-senescent cell origin. Senomorphics target these secreted products independently of their origin and without causing cell death.

Many promising preclinical and clinical trials have been conducted so far using senotherapeutic drugs [10,17,18]. The CANTOS and LoDoCo phase III clinical trials are the most promising ones among them, however, they did not change the clinical treatment strategy in practice due to concerns regarding the higher risk of infections [162,168,169]. There is still a need for further studies to find optimal treatment strategies and to confirm their efficacy, adverse off-target side-effects, and safety profiles. Some natural phytochemicals found in plants can exert anti-aging and anti-atherosclerotic effects by reducing oxidative stress, suppressing low-grade chronic inflammation, and inducing autophagy [144,145]. The most studied among them is resveratrol, which may have anti-aging and protective effects against atherosclerosis. This molecule might be considered as a primary preventive agent, however, the previous clinical studies were inconsistent regarding its beneficial effect. Importantly, growing evidence suggests a significant inverse association between frequent nut intake and cardiovascular disease supporting recommendations to include nuts as part of a healthy diet [156].

The treatment of atherosclerosis improved tremendously in the past decades including invasive and non-invasive strategies both in acute and non-acute clinical settings. However, the long-term clinical impact of treated arterial stenosis is still not negligible, suggesting the key role of atherosclerosis prevention. Management of modifiable cardiovascular risk factors (such as smoking, overweight, lifestyle, etc.) is an important step, nevertheless, a substantial residual risk of the development of relevant atherosclerosis still remains. The association of CHIP with cardiovascular risk was a milestone in understanding the new aspects of atherosclerosis. This highlights the importance of patient stratification beyond the use of traditional risk factors to define the patient population that could benefit from potential future CHIP-targeted treatment [10]. Senotherapeutics might open a new evolving era in the management of atherosclerosis. However, there is still work to be conducted in understanding cellular senescence and its complex interplay with other cells in atherosclerosis. Furthermore, it is essential to define and select the optimal senotherapy for the optimal patient population at the optimal time to achieve the best efficacy at the cost of the least side effects. Considering the worldwide burden of atherosclerosis, there is still an unmet need to develop further therapeutic strategies in atherosclerosis, especially in the prevention field, as preventing atherosclerosis is better than treating it.

## Data Availability

Not applicable.
